# Towards a Modern-Day Teaching Machine: The Synthesis of Programmed Instruction and Online Education

**DOI:** 10.1007/s40732-020-00415-0

**Published:** 2020-07-27

**Authors:** William B. Root, Ruth Anne Rehfeldt

**Affiliations:** 1 Department of Psychology and Counseling, Southeast Missouri State University, Cape Girardeau, MO 63701 USA; 2 The Chicago School of Professional Psychology, Chicago, IL USA

**Keywords:** Online education, Teaching machine, Programmed instruction, Automated instruction

## Abstract

The 21st century has seen rapid enrollment in online courses and environmental and biological determinates, such as COVID-19, that challenge how universities respond to education. However, this “new way of doing things” has empirical support from the past. Skinner (1968) laid out a science of teaching derived from operant conditioning principles and provided methods for adopting programmed instruction into what he termed a “teaching machine.” This series of investigations evaluated the validity of programmed instruction in online courses, as measured by quiz performance, the frequency of discussion posts, instructor time commitment, generalization, and student perceptions of the online modalities used. Results are discussed for the synthesis of programmed instruction and group learning towards a modern teaching machine.

Central to a technology of teaching is the role of the instructor. Unlike the Aristotelian and Platonic ideals of the teacher as *presenter* of instruction, Skinner (1968) emphasized their role as *the programmer* of instruction. Derived from his findings of the principles of reinforcement, Skinner built off of Pressey’s (1927) teaching machine to develop a systematic apparatus to program instruction. In this model, the instructor arranges carefully designed steps towards a mastery criterion, so small that “it can always be taken, yet in taking it the student moves somewhat closer to fully competent behavior” (Skinner, 1958, p. 970). Early applications of teaching machines generated promising demonstrations, utilized across primary, secondary, and university courses (Benjamin, 1988). However, there were several criticisms of automated education and the instructor's new role that led to slow adoption in mainstream applications.

Early critics remarked that machines cannot teach a “love” for the material (Margolis, 1963) or critical thinking (Rutherford, 2003), and that a “teacherless school" lacks the social components of learning, which is described as critical to the success of the student (Benjamin, 1988, p. 710). The picture of children sitting in isolation behind the teaching machine still resonates today. Instructors have also expressed concerns with automated instruction, with nearly two-thirds reporting views of inferior outcomes and the increased instructor time commitment (Allen & Seaman, 2016). Philosophers offer similar criticisms concerning automated instruction's adherence to determinism and the removal of the student and teacher's innate cognitive abilities. However, despite these criticisms, universities are beginning to adapt. In the field of applied behavior analysis (ABA), 33% of universities with verified course sequences do so through distance education, and 17% offer hybrid courses (Malkin, Rehfeldt, & Shayter, 2018). Online ABA courses offer several alternatives, such as a Massive Open Online Course (MOOC) (Rehfeldt, Jung, Aguirre, Nichols, & Root, 2016), online teacher consultation (Frieder, Peterson, Woodward, Crane, & Garner, 2009), parent training (Jang et al., 2012), and continuing education units offered through web-based training.

Several studies have compared student performance in online and on-campus courses (Diaz and Cartnal, 1999; Thompson, Klass, & Fulk, 2012). In the most notable study, Caywood and Duckett (2003) compared an online and on-campus teacher education course with 70 participants in both conditions. Results showed no statistical difference between groups, suggesting there is “no actual difference that can be measured in regards to learning” (Caywood & Duckett, 2003, p. 103). Online courses are further simulating on-campus settings with advances in webinar tools, text-based asynchronous learning opportunities, and discussion forums (Wang & Hsu, 2008). Instructors can now embed interactive components to the online classroom to improve student and instructor satisfaction. The synthesis of early applications of automated instruction, current pedagogies, and technologies appears to offer a modern application to the “teaching machine.”

Keller pioneered programmed instruction through the use of “test forms” that sequentially tested the material through self-paced time allotment, a criterion for mastery to move on to advanced “test forms,” lectures as “demonstrations” rather than “sources of critical information,” and proctors producing immediate feedback with a “personal-social aspect of the educational process” (Keller, 1968, p. 83). O’Grady, Reeve, Reeve, Vladescu, and Lake (2018) designed an automated protocol for teaching college students the visual analysis of single-subject designs. This program consisted of four module packets, similar to Keller’s test forms, that sequentially taught the course material. It is interesting that participants responded to questions that offered differing levels of prompting and feedback, which were then faded according to participant performance. Results showed that all participants mastered the material, demonstrated generalization to novel stimuli, and maintained accuracy in 1-month follow-ups.

Research combating the criticisms of social isolation associated with programmed instruction is necessary for its adoption. Indeed, there is a social component of the student experience, argued as crucial to the overall education the student receives (Dewey, 1923). Not only do students need to navigate the technical contingencies, but also the cultural contingencies to be successful in their future careers. The social component of education, once thought incompatible with online education, is now possible. Live online classrooms now offer microphones, cameras, polls, and chats to develop and facilitate real-time learning environments that approximate on-campus classrooms (Palloff & Pratt, 2002). Unfortunately, the same theories that plagued the adoption of Skinner’s (1968) *Technology of Teaching* and the reinforcers pulling the academic community away from its adoption are still salient today. However, in light of current contextual factors, namely the COVID-19 pandemic, higher education is forced to adapt. As an inductive approach to education, research must draw off of previous findings and begin to design the synthesis between contemporary and traditional approaches to online education, which, as we have found during this pandemic era, may no longer be optional.

The overall purpose of the combined studies was to evaluate the efficacy of programmed instruction in online courses. Experiment 1 directly compared the effects of on-campus delivered lectures and online delivered lectures on weekly quiz performance, percentage correct on within assessments forms, the frequency of questions asked, and generalization measures. Experiment 2 compared weekly quiz performance, the total number of minutes of instructor time commitment, generalization, and responses on the social validity questionnaire, following either lectures or module packets delivered exclusively online. Finally, Experiment 3 compared weekly quiz performance, generalization, and social validity following module packet + chat and lecture + discussion conditions.

## Experiment 1

### Method

#### Participants

Twenty-four undergraduate students enrolled in an introductory course in ABA participated in the current study. All participants had no or minimal courses in ABA.

#### Setting and Materials

Materials for each experimental condition included the assigned text, *Principles of Everyday Behavior Analysis* (Miller, 2006), Microsoft PowerPoint (2010) lectures, digital or paper copies of the within-lecture assessment forms, and personal laptop computers. All lectures were delivered by the primary researcher with 3 semesters experience teaching the course. All weekly quizzes took place in the on-campus lecture setting to ensure that participants did not have the opportunity to use outside materials. The weekly quizzes included 15 multiple-choice questions. The generalization quiz included 20 multiple-choice questions.

#### Variables, Response Measurement, and Reliability

The primary dependent variable was percentage correct on weekly quizzes. Each noncumulative weekly quiz consisted of 15 multiple-choice questions that assessed content covered in each week’s assigned chapters. The Desire2Learn (D2L) learner management system automatically graded all of the weekly quizzes. The secondary dependent variable was percentage correct on within-lecture assessment forms. There was a total of four fill-in-the-blank questions embedded in each session’s lecture slides, hand graded by the instructor. Throughout the study, participants did not receive copies of the slides.

The third dependent variable was the frequency of questions asked during each experimental session. Questions were scored as occurring if the participant asked a question related to the course material for that week. Questions were scored as not occurring if they were related to questions other than the course material, or related to previous content. The fourth dependent variable was the percentage correct on the generalization quiz. The final dependent variable was participant responses to the social validity questionnaire.

An independent observer collected trial-by-trial interobserver agreement (IOA) for 35% of within-lecture form assessments and frequency of questions asked. IOA for the within lecture assessment forms, for both conditions, was 100%. Trial-by-trial IOA for questions asked during on-campus and online sessions was 100%.

#### Design

An alternating treatments design compared on-campus and online lectures on weekly quiz scores, within lecture assessments, and frequency of questions asked during the lecture. Order of conditions was chosen using an online random number generator, with the stipulation that a condition not be conducted for more than two sessions in a row (Kazdin, 2010).

#### Procedure

##### On-campus lecture

The on-campus lecture condition took place on weeks 1, 3, 5, 6, and 9. Each week, there were two on-campus sessions delivered on Tuesday and Thursday. On Tuesdays, participants came to the on-campus setting for a 75-min PowerPoint instructor-led lecture that included 30 to 40 slides and covered one chapter of the assigned text. The lecture followed the same format each week. First, there was a recap of the previous material, then definitions of weekly content, and finally graphical representations and clinical vignettes. Each lecture included a total of four within-lecture assessment questions that required participants to answer fill-in-the-blank questions related to the content covered in the lecture for that day. On Thursdays, participants received a 35-min PowerPoint instructor-led lecture covering a new chapter, delivered in the same format as Tuesday’s lecture. Following the lecture, participants received a 10-min break before completing a 30-min, noncumulative weekly quiz.

##### Online lecture

The online lecture conditions took place on weeks 2, 4, 7, 8, and 10. There were two online lectures delivered each week that took place on Tuesdays and Thursdays. The online lecture utilized the same format as the on-campus lecture. Following Thursday’s lecture, participants had 10 min to come to the on-campus setting to engage in the weekly quiz. Weekly quizzes were conducted in the on-campus setting to decrease the likelihood of using outside materials.

##### Generalization quiz

The generalization quiz took place on week 11, following all formal experimental conditions. Participants met in the on-campus setting and were required to complete a 20-question cumulative multiple-choice quiz, with 10 questions resembling content covered in each condition. Participants received 75 min to complete the generalization quiz.

##### Social Validity

Social validity assessed the participants’ opinion on both instructional modalities, ease of use, quality of instruction for both experimental conditions, the likelihood of enrolling and recommending a course delivered exclusively online, and the degree to which the participants recognized the value in investigations of online courses.

## Experiment 1

### Results

#### Weekly Quiz Scores

##### On-campus lecture

**Fig. 1:**
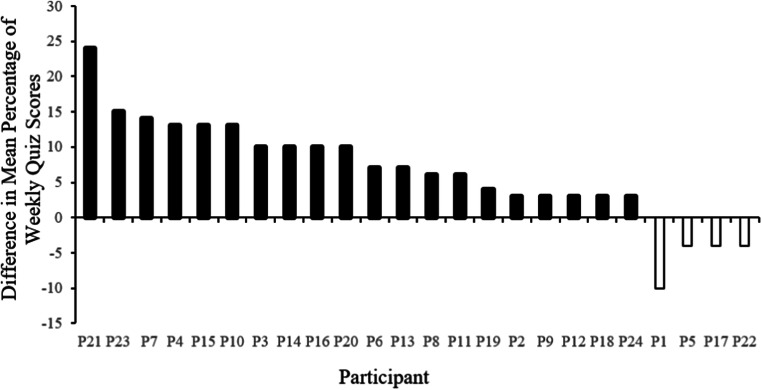
Mean difference in weekly quiz scores in Experiment 1. Each bar represents a single participant. The solid black bars represent participants who scored with a higher mean difference following online lectures and open bars represent participants who scored with a higher mean difference following on-campus lectures

Figure 1 represents the mean difference in weekly quiz scores. Each bar represents a single participant. The open bars represent participants who scored with a higher mean difference following on-campus lectures. Mean quiz scores following on-campus delivered lectures ranged from 90% to 56%, with an overall mean of 77.2% correct on weekly quizzes.

##### Online lecture

Figure 1 represents the mean difference in weekly quiz scores. Each bar represents a single participant. The solid black bars represent participants scoring with a higher mean difference following the online lecture condition. Mean quiz scores following online lectures ranged from 96% to 66%, with an overall mean of 83.7% correct.

#### Within-Lecture Assessment Form

The mean percentage correct following online lectures ranged from 98% to 94% correct, with an overall mean of 96% correct. The mean percentage correct following on-campus delivered lectures ranged from 98% to 67% correct, with an overall mean of 93% correct.

#### Frequency of Questions Asked

The mean frequency of questions asked during online lectures ranged from 4 to 0, with an overall mean of 2 questions asked. Mean frequency during on-campus lectures ranged from 3 to 0, with an overall mean of 1 question asked.

#### Generalization Test

For questions covered in on-campus lectures, the mean percentage correct was 70%. For questions covered in online lectures, the mean percentage correct was 72%. Overall, there was a difference in percentage correct of 2%, with a higher observed difference for questions asked during online lecture conditions

#### Social Validity

The mean score reported for question 1 was 4 (range: 4–5), the mean score for question 2 was 4 (range: 4–5). The mean score reported for question 3 was 4 (range: 4–5), the mean score for question 4 was 4 (range: 4–5). The mean score reported for question 5 was 4 (range: 4–5), the mean score for question 6 was 4 (range: 4–5).

## Experiment 1

### Discussion

The results of Experiment 1 further support the potential utility of lectures delivered online, when compared to traditional on-campus lectures (Dutton, Dutton, & Perry, 2001). Overall, 20 out of 24 participants performed with higher quiz accuracy during quizzes associated with online lecture weeks, with 10 of the 24 participants performing a full letter grade higher. The results of the within lecture assessment forms showed a difference in means between the two conditions of 3%, with higher mean performance observed in online lecture conditions. Although not a substantial difference between the two conditions, these results highlight their importance. Reeves (2000) comments that automated instruction can provide “performance assessment data" (p. 108) that can key the instructor to areas to spend time on during a review before the quizzes or during future lectures. Experiment 1 also revealed a low frequency of questions asked during either the online or the on-campus lectures. Overall, there was a difference mean frequency of 1 question, with higher mean of questions asked observed in online lecture conditions. Often, the cultural contingencies in the on-campus classroom, such as proximity to desk mate, under-breath laughter, and social cues, may serve to punish questions asked. Those contingencies may not be as salient in the online context.

A possible limitation for the frequency of questions asked is that data were not collected on participant attendance, so it is unclear if each week there was a different number of participants to ask questions. Future studies could collect this type of data to ensure consistency throughout the study. Another possible limitation to Experiment 1 is that each experimental condition correlated with different content learned throughout the course. A critique of these measures is that some weeks could be "harder" than others, which may decrease the internal validity of the findings (Caywood & Duckett, 2003). Although the randomized conditions consistent with the alternating treatments design controls for this type of threat to internal validity, future research could strengthen the findings with studies that use one chapter, shorter duration than an entire semester, or counterbalancing chapters by group.

Experiment 2 compared online module packets and online lectures to further to identify programmed instruction's effectiveness in online applications. In this way, Experiment 2 compared weekly quiz performance, the total number of minutes of instructor time commitment, generalization, and responses on the social validity.

### Experiment 2

### Method

#### Participants

Eight participants enrolled in a graduate ethics course in behavior analysis participated in the current study. All participants had no or minimal courses in behavioral ethics.

#### Online Classroom

All experimental conditions were done exclusively in online learner management systems, D2L and Adobe Connect Meeting (2017).

#### Setting and Materials

Materials for each experimental condition included the assigned text, *Ethics for Behavior Analysts, 3*^*rd*^ *Edition* (Bailey & Burch, 2016), and personal laptop computers. Weekly quizzes comprised of a total of 10 multiple-choice questions. The generalization test included a total of 20 multiple-choice questions. During online lecture conditions, the weekly online lecture materials included 30 to 40 slides that presented the material covered during sessions. During the module packet conditions, materials included weekly module packets. In particular, the module packets served to teach successive approximations to mastery of the weekly content, students could access them at any time throughout the week to create a self-paced learning environment, and the use of the automatic grading function in D2L allowed the module packets to produce immediate student feedback. Each week, there were a total of three weekly module packets.

#### Variables, Response Measurement, and Reliability

The primary dependent variable was percentage correct on weekly quizzes. The secondary dependent variable was the total minutes of instructor time commitment. During online lecture conditions, the instructor logged the total minutes spent preparing the lecture. Total minutes of instructor time commitment delivering the lecture was included as part of the development of course materials during the online lecture condition. During the module packet conditions, the total minutes spent developing the module packets were logged for the week. Time spent during individual student meetings was not included during both experimental conditions. Although the instructor had taught this course in previous semesters, he did not use previous course materials or slides to control for a possible confound in instructor time commitment. The third dependent variable was the percentage correct on the generalization quiz. The final dependent variable was participant preference.

IOA was collected on total minutes of instructor time commitment. Before developing the weekly materials associated with each experimental condition, the instructor started a timer when he began preparing and stopped the timer when he had completed preparing the course materials. There was no set day or time frame for the instructor to develop the weekly course materials, only that they were required to be completed a week before the weekly session. However, the designated time for IOA sessions was scheduled. For 35% of both experimental conditions, a secondary observer was scheduled with a specific time to observe total minutes of instructor time commitment to generate IOA. Trial-by-trial IOA for total hours of instructor time commitment during module packet conditions was 100%.

#### Design

An alternating treatments design compared online lecture conditions and module packet conditions on percentage correct on weekly quizzes, total minutes of instructor time commitments, and generalization to novel questions.

#### Procedure

##### Online lecture

The online lecture took place on Wednesday evenings, from 6:00 pm to 7:00 pm CT. The lecture included 30 to 40 slides that covered weekly course material. On Fridays, anytime from 8:00 am to 11:59 pm CT, participants were required to engage in a 10-question, 30-min, multiple-choice quiz.

##### Module packets

During module packet conditions, there were a total of three module packets available on Monday from 8:00 am to Thursday at 11:59 pm CT. Module packets consisted of 10 multiple questions. Module Packet 1 tested name-to-definition, Module Packet 2 tested definition-to-name, and Module Packet 3 tested name-to-clinical vignette. Following the module packet submission, participants were provided with feedback on incorrect or correct responses by a computer-generated statement of "incorrect" or "correct" for each question. If an answer was incorrect, participants were required to complete the module packet until all responses were scored as correct to move on to the next module packets. On Fridays, anytime from 8:00 am to 11:59 pm CT, participants were required to take the weekly quiz.

#### Social Validity

The Social Validity Questionnaire evaluated participant preference for both instructional modalities, whether participants felt isolated from peers and instructor during module packet weeks, level of preparedness for the weekly quizzes associated with each condition, and whether they would take a course similar to module packet weeks again.

## Experiment 2

### Results

#### Weekly Quiz Scores

##### Online lecture

**Fig. 2:**
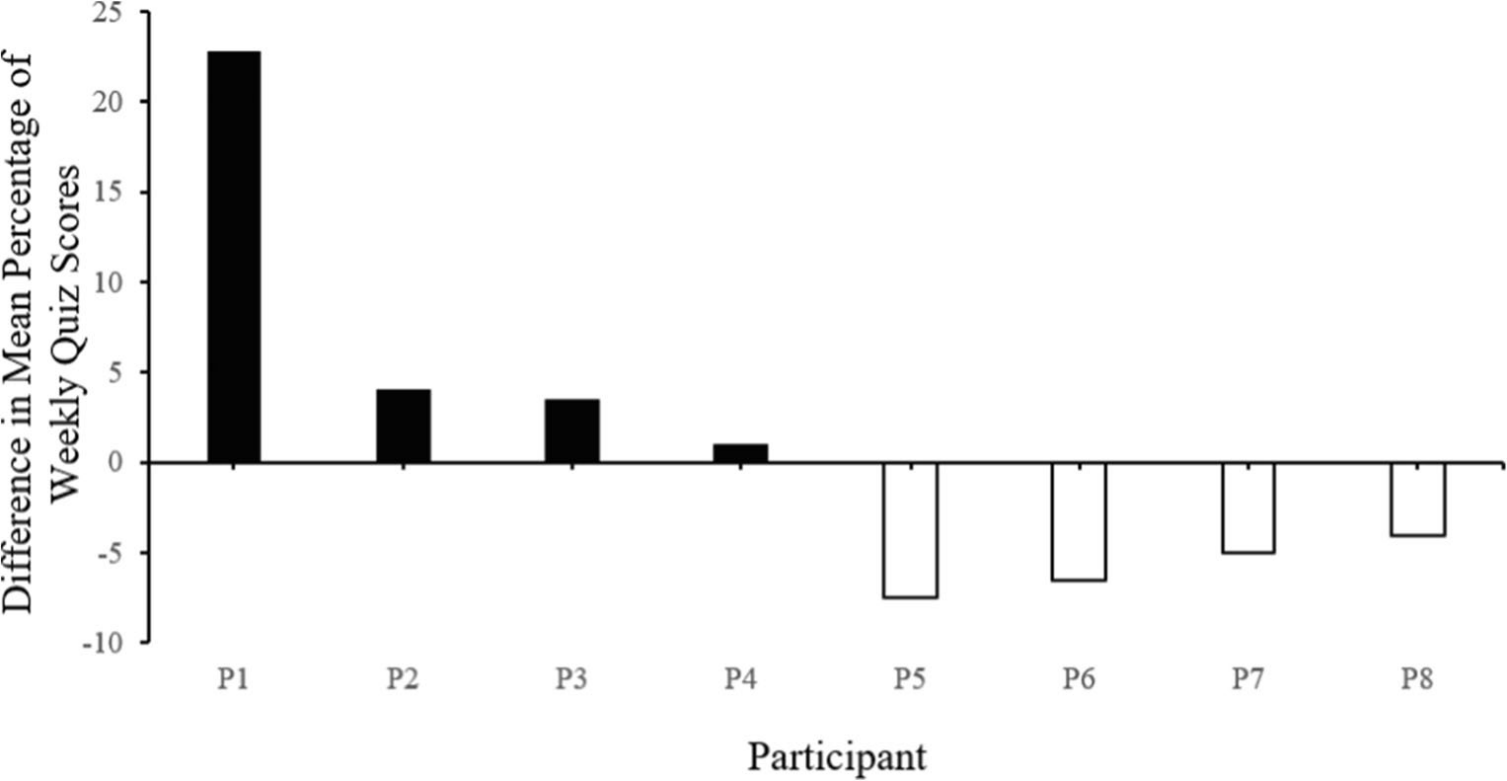
Mean difference in weekly quiz scores in Experiment 2. Each bar represents a single participant. The solid black bars represent participants who scored with a higher mean difference following module packet conditions and open bars represent participants who scored with a higher mean difference following online lecture conditions

Figure 2 represents the mean difference in weekly quiz scores. Each bar represents a single participant. The open bars represent participants that scored with a higher mean difference following the online lecture conditions. Mean quiz scores following online lectures ranged from 71% to 100%, with an overall mean of 89% correct on weekly quizzes.

##### Module packets

Figure 2 represents the mean difference in weekly quiz scores. Each bar represents a single participant. The solid black bars represent participants scoring with a higher mean difference following the module packet conditions. Mean quiz scores following module packets ranged from 85% to 95%, with an overall mean of 91% correct on weekly quizzes.

#### Instructor Time Commitment

During initial online lecture conditions, the instructor time commitment was 96 min, increasing to 114 min by week 4, and decreasing to 92 min by the final session. During initial module packet conditions, the instructor time commitment was 140 min, decreasing to 126 min, increasing to 144 min, and finally decreasing to 126 min by the final session.

#### Generalization Test

For questions covered in module packets sessions, the mean percentage correct was 98%. For questions covered in online lecture sessions, the mean percentage correct was 94%. Overall, there was a difference in percentage correct of 4%, with higher levels of generalization observed in the module packet condition.

#### Social Validity

The mean score reported for question 1 was 4 (range: 4–5), The mean score reported for question 2 was 4 (range: 3–5), the mean score reported for question 3 was 4 (range: 4–5. The mean score reported for question 4 was 4 (range: 3–5), the mean score reported for question 5 was 4 (range: 4–5). The mean score reported for question 6 was 4 (range: 3–5), the mean score reported for question 7 was 4 (range: 4–5).

## Experiment 2

### Discussion

Overall, half of the participants in Experiment 2 performed with greater accuracy during the online lecture and half of the participants during the module packet conditions. These results are consistent with a critical conceptual stance of Skinnerian teaching methodologies. That is, the data will guide the most effective program of instruction for each learner (Skinner, 1968). For example, at the beginning of the semester, the instructor could vary lectures and module packets, and provide instruction based on the student’s highest-performing conditions. The opportunity to provided individualized instruction is often not feasible in the on-campus setting (Diaz and Cartnal, 1999). The results of Experiment 2 are consistent with a common concern for instructors of the increased time commitment for online courses (Allen & Seaman, 2016), with an average of 34 min a week longer during module packet conditions. However, the majority of time was spent on programming the module packets into the online database, which will not require the same amount of time in future applications. The instructor's time can now focus on refining the module packets, such as including more current behavior analytic teaching arrangements like precision teaching (Lindsley, 1992), interteaching, and stimulus equivalence protocols (Walker & Rehfeldt, 2012).

The module packets met the Skinnerian criterion for programmed instruction in four ways. The first method "is concerned with generating new and complex patterns or 'topographies' of behavior" (Skinner, 1984, p. 430). The conditional discriminations of the module packet questions met this first criterion, efficiently designed into the online learner management system. The second method of programming "is used to alter temporal or intensive properties of behavior" (p. 432). Each module packet taught new responses reinforced through successful completion, which then allowed the learner to move to the next module packet and meet the third method of programming, which "is concerned with bringing behavior under the control of stimuli" (p. 432). The fourth method of programming "has to do with maintaining behavior under infrequent reinforcement" (p. 435). The reinforcer was a passing grade at the end of a semester. The module packets allowed students to be reinforced throughout the course to keep them engaged and thriving throughout the program. As Skinner (1965) recounts, "Maintaining a high level of activity is one of the more important parts of programmed instruction" (p. 435).

Experiment 3 examined the effect of including a group learning component to the module packet condition (chat) and a discussion component to the lecture condition. In this way, Experiment 3 compared the percentage correct on weekly quizzes, generalization, and the social validity, following module packet + chat and lecture + discussion conditions.

## Experiment 3

### Method

#### Participants

Ten participants enrolled in a graduate course in behavior analysis participated in the current study. All participants had no or minimal courses in ethics for behavior analysts.

#### Online Classroom

All experimental conditions were implemented exclusively in online learner management systems, Desire2Learn (D2L), and Adobe Connect Meeting 2017.

#### Materials

The online lecture + discussion materials were identical to those used in Experiment 2, with the inclusion of weekly online discussion forums on D2L. The module packets + chat materials were identical to the module packets used in Experiment 2, with the inclusion of a weekly chat on D2L. There was a total of 10 weekly quizzes.

#### Variables, Response Measurement, and Reliability

The primary dependent variable was percentage correct on weekly quizzes. The secondary dependent variable was the participant preference for online lecture + discussion conditions and module packet + chat conditions. The final dependent variable was the percentage correct on the generalization test.

#### Design

An alternating treatments design compared online lecture + discussion conditions and module packet + chat conditions on percentage correct on weekly quizzes and the generalization condition.

#### Procedure

##### Online lecture + Discussion condition

The online lecture took place on Thursdays, from 7:00 pm to 8:00 pm CT, and was delivered in a manner similar to that of Experiment 2. Participants were also required to respond to a total of two weekly discussion forums. Discussion Forum 1 was available on Monday at 8:00 am and closed by Tuesday at 11:59 pm CT. Discussion Forum 2 was available from Wednesday at 12:00 am and closed by Thursday at 11:59 pm CT. Participants were required to respond to one instructor provided question, provide a question, and respond to one of their classmates' responses during each Discussion Forum. The role of the instructor was to provide general feedback to the questions posed by the participants. To reduce the instructor time commitment, the instructor viewed the discussions boards once at 9:00 am and once at 5:00 pm CT, randomly selected one participant response by selecting a participant name out of a box, and provided a two-sentence response to the participant's questions. Sentences provided feedback concerning: 1) if the discussion was on track, and 2) where to look in the reading or lecture to find the answer. Finally, participants were required to take a 20-question, 30-min, noncumulative multiple-choice quiz that covered the weekly material. Participants were able to access and take the quiz anytime on Friday at 8:00 am to Friday at 11:59 pm CT.

##### Module packet + Chat

Module packets were conducted similarly to those used in Experiment 2. Participants were also required to participate in a 60-min live chat. The online chat took place on Thursdays, from 7:00 pm to 8:00 pm CT. During chat sessions, the instructor presented each term covered in Module packet 1, 2, and 3 in sequential order. Each participant had the opportunity to either ask a question, provide an example from her career, or provide the definition. If none of the participants responded to the question, the instructor would randomly select a participant and ask them to define the term. Finally, participants were required to take a 20-question, 30-min, multiple-choice quiz available on Friday from 8:00 am to 11:59 pm CT.

#### Social Validity

A Social Validity Questionnaire assessed the participant's opinion on the different instruction modalities, student perception of isolation during the module packet + chat condition, quality of instruction, preparation, and whether students would recommend module packet + chat courses in the future.

## Experiment 3

### Results

#### Weekly Quiz Scores

##### Online lecture + Discussion boards

**Fig. 3:**
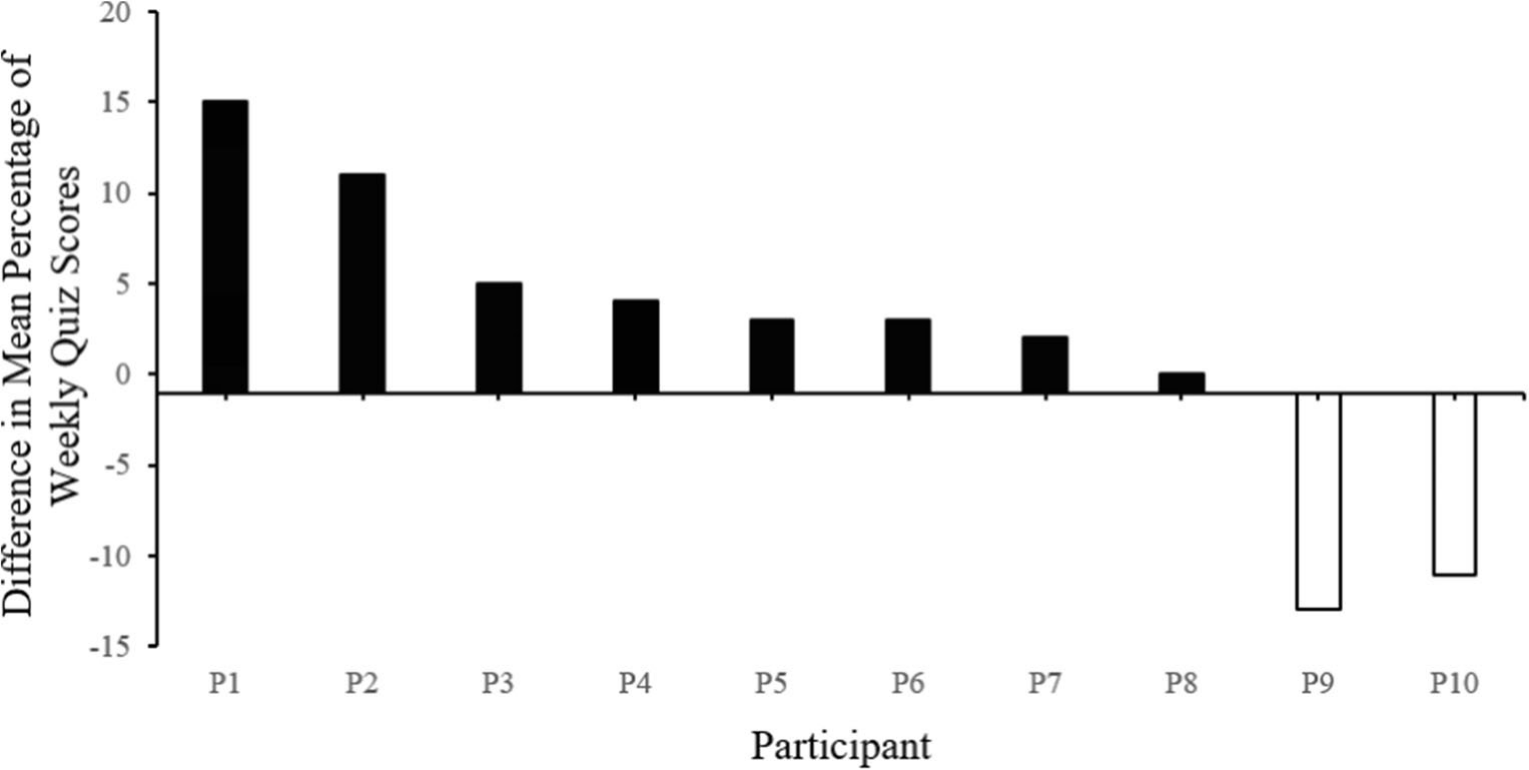
Mean difference in weekly quiz scores in Experiment 3. Each bar represents a single participant. The solid black bars represent participants who scored with a higher mean difference following module packet + chat conditions and open bars represent participants who scored with a higher mean difference following online lecture + discussion conditions

Figure 3 represents the mean difference in weekly quiz scores. Each bar represents a single participant. The open bars represent participants that scored with a higher mean difference following online lecture + discussion conditions. Mean quiz scores following online lectures + discussion boards ranged from 73% to 100%, with an overall mean of 89% correct on weekly quizzes.

##### Module packets + Chat

Figure 3 represents the mean difference in weekly quiz scores in Experiment 3. Each bar represents a single participant. The solid black bars represent participants scoring with a higher mean difference following module packet + chat conditions. Mean quiz scores following module packets + chat ranged from 82% to 100%, with an overall mean of 91% correct on weekly quizzes.

#### Generalization Test

For questions covered in module packet + chat, the mean percentage correct was 97%. For questions that covered in online lectures + discussion boards, the mean percentage correct was 93%. Overall, there was a difference in percentage correct of 4%, with the mean percentage correct in questions that pertained to material covered in the online lecture conditions.

#### Social Validity

The mean score reported for question 1 was 4 (range: 4–5), the mean score reported for question 2 was 4 (range: 4–5). The mean score reported for question 3 was 4 (range: 3–5), the mean score reported for question 4 was 4 (range: 3–5). The mean score reported for question 6 was 4 (range:3–5), the mean score reported for question 7 was 4 (range: 3–5).

## Experiment 3

### Discussion

With 7 out of 10 participants performing higher following the module packet + chat conditions, the results of Experiment 3 are consistent with previous literature that suggests a synthesis of group learning and programmed instruction in online learning arrangements (Diaz & Cartnal, 1999; Frieder et al., 2009; Malkin et al., 2018). Generalization of field-specific repertoires outside of the university is an obstacle facing education delivered exclusively online (Alexander, Lignugaris/Kraft, & Forbush, 2007). When analyzing individual differentiation between the two conditions, 6 out of 10 participants performed a letter grade higher following module packet + chat sessions. The difference between generalization effects could be a function of the group learning opportunities that occurred during the chat component. For example, the opportunity for an in-depth discussion on key concepts of the curriculum could have been a contributing variable to the generalization effects.

One exciting component of group learning in a chat is the opportunity to develop a repertoire of verbal behavior concerning the subject of study. Often in the traditional online classroom, students are primarily exposed to written responses or selection-based responses to demonstrate mastery. However, ways to develop written and vocal responses in the university setting have been studied and applied in the behavior analytic classroom (Walker & Rehfeldt, 2012). Boyce and Hineline (2002) suggested that the use of group learning components enhances learner outcomes and the "User-Friendliness" of behavior analytic methods of instruction.

Although the results are promising for the inclusion of a group learning component into both programmed instruction and online learning arrangement, there are several limitations worth mentioning. First, given that each condition incorporated a different type of group learning arrangement, asynchronous discussion, and synchronous chat, this distinction may not provide an accurate comparison. Future studies could address this limitation by directly comparing chat and discussion alone on participant performance.

## General Discussion

The combined studies add to the body of literature, which continues to demonstrate that online lectures can simulate traditional on-campus lectures (Dutton et al., 2001) and the use of technological advances to incorporate a group learning component to automated instruction. Experiment 1 demonstrated the utility of online lectures to simulate traditional lectures delivered on-campus. Experiment 2 asked the question, "What role does the instructor play?" by comparing self-paced module packets without instructor presence and online lectures. Although the results of Experiment 2 demonstrated similar mean responding during both conditions, they further highlight the need for individual instruction. Experiment 3 embedded a group learning component to Skinnerian programmed instruction through the use of a chat. The inclusion of a group learning component to automated instruction supports the conclusion that this synthesis may be an effective method for delivering online university courses (Saville, Zinn, & Elliott, 2005; Schneider, Kerwin, Frechtling, & Vivari, 2002). Although the results of Experiment 1, 2, and 3 are promising for online education as a whole, there are several important implications in the field of behavior analysis.

Traditional ideologies are still present in mainstream education and have led many credentialing boards to suggest several hours of student engagement in distance education to mimic instruction hours in traditional on-campus lecture halls. For example, in the field of behavior analysis, instructional time must be equivalent to on-campus classroom hours. For the board-certified behavior analyst (BCBA) course requirements, there must be a total of 270 verified instructional hours allocated through the graduate term (Behavior Analyst Certification Board, 2016). However, university-imposed deadlines and time constraints may not be congruent with the theoretical underpinnings of programmed instruction. For example, Barlow (1960) utilized programmed instruction and commented on the brevity of the course. The instructor was able to teach the entire summer’s material in one day. However, as stated by the credentialing board, unless instructional time met the hour-based criteria, this would not have counted towards a verified course sequence. For Skinner (1968), the instruction was not bound by time, but by mastery.

Even further, education is not an intrinsic ability of the instructor, a student, or even a place. It is a process of the contingencies of learning (Skinner, 1968). As the 21st century brings about unprecedented barriers to the university's traditional role as a whole, research into effective methods for designing online formats is paramount. The COVID 19 pandemic has revealed several flaws in the brick-and-mortar, chalkboard lectures of the past. Programmed instruction led the way for a science of teaching, and online learning formats offer a modern, timely application. The utility and ease of online education to deliver individualized, programmed, self-paced instruction cannot be understated. It would be a shame for online education to not build off of an old machine.

## References

[CR1] Alexander M, Lignugaris/Kraft B, Forbush D (2007). Online mathematics methods course evaluation: Student outcomes, generalization, and pupil performance. Teacher Education & Special Education.

[CR2] Allen IE, Seaman J (2016). *Online report card: Tracking online education in the United States*.

[CR3] Bailey JS, Burch MR (2016). *Ethics for behavior analysts*.

[CR4] Barlow JA (1960). Conversational chaining in teaching machine programs. Psychological Reports.

[CR5] Behavior Analyst Certification Board. (2016). *VCS handbook*. Retrieved September 9, 2019, from http://info.bacb.com/o.php?page=100358. https://www.bacb.com/wp-content/uploads/180326-VCS-Handbook.pdf

[CR6] Benjamin LT (1988). A history of teaching machines. American Psychologist.

[CR7] Boyce TE, Hineline PN (2002). Interteaching: A strategy for enhancing the user-friendliness of behavioral arrangements in the college classroom. The Behavior Analyst.

[CR8] Caywood K, Duckett J (2003). Online vs. on-campus learning in teacher education. Teacher Education & Special Education.

[CR9] Dewey J (1923). *Democracy and education: An introduction to the philosophy of education*.

[CR10] Diaz DP, Cartnal RB (1999). Students' learning styles in two classes: Online distance learning and equivalent on-campus. College Teaching.

[CR11] Dutton J, Dutton M, Perry J (2001). Do online students perform as well as lecture students?. Journal of Engineering Education.

[CR12] Frieder JE, Peterson SM, Woodward J, Crane J, Garner M (2009). Teleconsultation in school settings: Linking classroom teachers and behavior analysts through web-based technology. Behavior Analysis in Practice.

[CR13] Jang, J., Dixon, D. R., Tarbox, J., Granpeesheh, D., Kornack, J., & de Nocker, Y. (2012). Randomized trial of an eLearning program for training family members of children with autism in the principles and procedures of applied behavior analysis. *Research in Autism Spectrum Disorders, 6*(2), 852–856. 10.1016/j.rasd.2011.11.004

[CR14] Kazdin AE (2010). *Single-case research designs*.

[CR15] Keller FS (1968). Good bye teacher. Journal of Applied Behavior Analysis.

[CR16] Lindsley OR (1992). Precision teaching: Discoveries and effects. Journal of Applied Behavior Analysis.

[CR17] Malkin A, Rehfeldt RA, Shayter AM (2018). An investigation of the efficacy of asynchronous discussion on students’ performance in an online research method course. Behavior Analysis in Practice.

[CR18] Margolis RJ (1963). Do teaching machines really teach. Redbook.

[CR19] Miller K (2006). *Principles of everyday behavior analysis*.

[CR20] O’Grady AC, Reeve SA, Reeve KF, Vladescu JC, Lake CMJ (2018). Evaluation of computer-based training to teach adults visual analysis skills of baseline-treatment graphs. Behavior Analysis in Practice.

[CR21] Palloff, R. M., & Pratt, K. (2002). *Lessons from the cyberspace classroom: The realities of online teaching*. San Francisco, CA: Wiley.

[CR22] Pressey SL (1927). A machine for automatic teaching of drill material. School & Society.

[CR23] Reeves, T. C. (2000). Alternative assessment approaches for online learning environments in higher education. Journal of Educational Computing Research, 23(1), 101–111. 10.2190/GYMQ-78FA-WMTX-J06C

[CR24] Rehfeldt RA, Jung HL, Aguirre A, Nichols JL, Root WB (2016). Beginning the dialogue on the e-transformation: Behavior analysis’ first massive open online course (MOOC). Behavior Analysis in Practice.

[CR25] Rutherford A (2003). BF Skinner's technology of behavior in American life: From consumer culture to counterculture. Journal of the History of the Behavioral Sciences.

[CR26] Saville BK, Zinn TE, Elliott MP (2005). Interteaching versus traditional methods of instruction: A preliminary analysis. Teaching of Psychology.

[CR27] Schneider SJ, Kerwin J, Frechtling J, Vivari BA (2002). Characteristics of the discussion in online and face-to-face focus groups. Social Science Computer Review.

[CR28] Skinner BE (1958). Teaching machines. Science.

[CR29] Skinner BF (1965). Review lecture: The technology of teaching. Proceedings of the Royal Society of London: Series B Biological Sciences.

[CR30] Skinner BF (1968). *The technology of teaching*.

[CR31] Skinner BF (1984). The shame of American education. American Psychologist.

[CR32] Thompson JR, Klass PH, Fulk BM (2012). Comparing online and face-to-face presentation of course content in an introductory special education course. Teacher Education & Special Education.

[CR33] Walker BD, Rehfeldt RA (2012). An evaluation of the stimulus equivalence paradigm to teach single-subject design to distance education students via Blackboard. Journal of Applied Behavior Analysis.

[CR34] Wang, S. K., & Hsu, H. Y. (2008). Use of the webinar tool (Elluminate) to support training: The effects of webinar-learning implementation from student-trainers’ perspective. *Journal of Interactive Online Learning, 7*(3), 175–194.

